# Causal and common risk pathways linking childhood maltreatment to later intimate partner violence victimization

**DOI:** 10.1038/s41380-024-02813-0

**Published:** 2024-11-02

**Authors:** Patrizia Pezzoli, Jean-Baptiste Pingault, Thalia C. Eley, Eamon McCrory, Essi Viding

**Affiliations:** 1 Division of Psychology and Language Sciences, University College London (UCL), London, UK; 2 Social, Genetic & Developmental Psychiatry Centre, Institute of Psychiatry, Psychology & Neuroscience, King’s College London, London, UK

**Keywords:** Genetics, Psychology, Psychiatric disorders

## Abstract

Childhood maltreatment and intimate partner violence (IPV) victimization are major psychiatric risk factors. Maltreatment substantially increases the likelihood of subsequent IPV victimization, but what drives this association is poorly understood. We analyzed retrospective self-reports of maltreatment and IPV victimization in 12,794 participants (58% women, 42% men) from the Twins Early Development Study at ages 21 and 26 using quantitative genetic methods. We estimated the etiological influences common to maltreatment and IPV, and the effect of maltreatment on IPV beyond such common influences. Participants who reported childhood maltreatment ( ~ 7% of the sample) were 3 times more likely than their peers to also report IPV victimization at age 21, 4 times more likely at 26. The association between maltreatment and IPV was mostly due to environmental influences shared by co-twins (42–43%) and genetic influences (30–33%), as well as nonshared environmental influences (25–27%). The association between maltreatment and IPV was similar for women and men, but its etiology partly differed by sex. Maltreatment had a moderate effect on IPV in phenotypic models (*β* = 0.25–0.30), decreasing to a small-to-moderate range in causally informative models accounting for their common etiology (*β* = 0.15–0.21). Risk factors common to maltreatment and IPV victimization are largely familial in origin, environmental and genetic. Even considering common risk factors, experiencing maltreatment may be causally related to subsequent IPV victimization. Interventions promoting safe intimate relationships among young adults exposed to maltreatment are warranted and should address family-level environmental risk and individual-level risk shaped by genetics.

## Introduction

Globally, about 20% of adults report experiencing at least one form of maltreatment by parents or caregivers before the age of 16, with prevalence rates varying across types of maltreatment, such as abuse and neglect [1–4]. Childhood maltreatment (hereafter, maltreatment) is a major risk factor for psychiatric disorders, substantially adding to the global burden of disease [5, 6]. Furthermore, individuals exposed to maltreatment face a higher risk of experiencing interpersonal violence later in life compared to their peers – a phenomenon known as “revictimization” [7–11]. A critical form of revictimization after maltreatment is intimate partner violence (IPV), with individuals exposed to maltreatment being 3–6 times more likely to experience IPV victimization from age 16 than their peers [12, 13]. It has been estimated that about 20–30% of adults experience some form of IPV victimization throughout their lives [14, 15], but this prevalence can rise up to 77% among those with a history of maltreatment [16]. IPV occurs in heterosexual and homosexual relationships to a similar extent [17–19]. Some studies report higher prevalence rates of IPV victimization in women than men, particularly more sexual abuse [20–22], while others report comparable estimates, particularly for physical abuse [23, 24]. What is noteworthy is that individuals exposed to maltreatment face heightened risk of IPV victimization and that this is true for different sexual orientations and genders [12, 25–27]. Akin to maltreatment, IPV victimization increases psychiatric risk [28, 29]. Moreover, experiencing both maltreatment and IPV has a more detrimental impact on mental health than either experience alone [30, 31]. In light of this evidence base, elucidating the underlying processes linking maltreatment to subsequent IPV victimization is critical to develop effective preventative interventions.

### Possible pathways from childhood maltreatment to intimate partner violence victimization

**Fig. 1:**
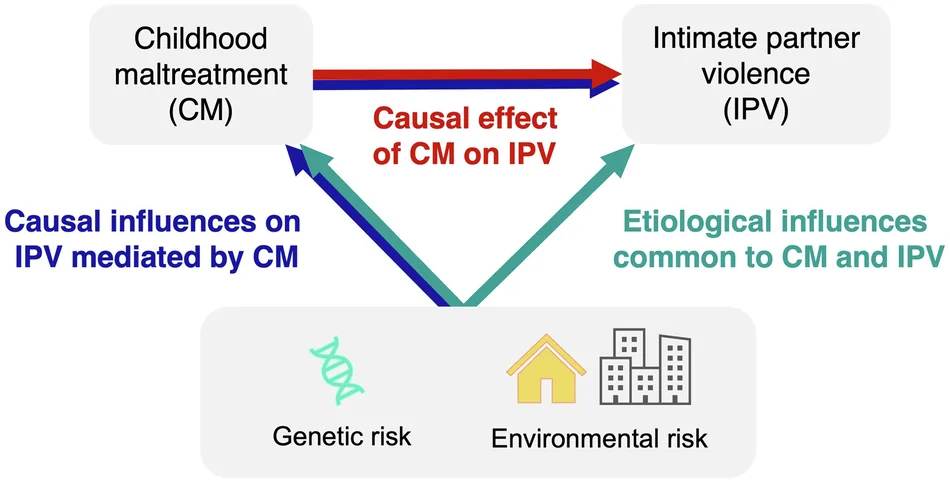
Possible pathways underlying the association between childhood maltreatment and subsequent intimate partner violence.

The prevailing hypothesis regarding the association between maltreatment and subsequent IPV victimization proposes that maltreatment initiates processes that are, in turn, causally related to entering abusive relationships (Fig. 1, red and blue paths). This is in line with state dependence theories of revictimization, which postulate that victimization can lead to changes in an individual that increase their risk of revictimization [32, 33]. Cross-sectional population-based data hints at a number of psychological (e.g. emotional dysregulation [34, 35]) and social processes (e.g., relationship quantity and quality [36, 37]) that partly, but modestly, mediate the longitudinal association between maltreatment and IPV victimization. However, formal examinations of putative causal effects of maltreatment on subsequent IPV victimization are lacking.

An alternative hypothesis postulates that the association between separate victimization events – such as between maltreatment and IPV victimization – is non-causal and, rather, driven by shared time-stable risk factors (green path). This is consistent with the population heterogeneity theory of revictimization [32, 38]. Evidence that maltreatment and IPV victimization share a number of common risk factors lends support to this hypothesis. For example, population-based studies indicate that environmental risk factors like socioeconomic disadvantage place individuals at risk of experiencing both maltreatment [39, 40] and IPV [41, 42]. However, systematic reviews and meta-analyses indicate that psychological vulnerabilities have an even greater effect size than environmental factors on the risk of maltreatment [43, 44] and IPV [45–47]. Since similar environmental and psychological factors may increase risk of experiencing both maltreatment and IPV, the putative causal effect of maltreatment on IPV beyond their shared etiology should be examined.

### The utility of genetically informed designs to disentangle risk pathways to revictimization

Extant research suggests that both causal pathways and common risk factors are likely to contribute to the association between maltreatment and subsequent IPV victimization. Studies using phenotypic methods like structural equation modeling and multivariable logistic regression indicate a persisting effect of maltreatment on subsequent IPV victimization, even when accounting for specific individual- (e.g., aggressive behavior) and family-level factors (e.g., social deprivation) known to confer risk to both experiences [48–50]. However, phenotypic methods may overestimate the effect of maltreatment on subsequent IPV victimization because they can only account for a finite number of common risk factors. Genetically informed designs like the twin design provide a quasi-experimental framework to estimate the common etiology between maltreatment and IPV, and to examine their causal relationships beyond such common etiology, without measuring specific common risk factors [51, 52]. Studies using genetically informed designs indicate, for instance, that maltreatment has a small additional effect on mental health problems beyond its common etiology with mental health problems [53, 54].

The twin design has been employed to investigate the etiology of maltreatment and IPV victimization separately, by comparing identical (monozygotic) and fraternal (dizygotic) twins, who differ in their genetic resemblance. Identical twins share 100% of their segregating genes, while fraternal twins share about 50%, and both types of twins typically grow up in the same environment. Despite being raised in the same household, however, co-twins can have different experiences, including exposure to maltreatment [55]. If identical twins show greater similarity in experiences compared to fraternal twins, this indicates that genetic factors may play a role in such experiences. Twin studies have shown that genetic factors and environmental exposures that make co-twins dissimilar (“nonshared” environment) each explain a moderate-to-large proportion of individual differences in maltreatment (40–60%), with environmental exposures that contribute to the similarity between co-twins (“shared” environment) having a small-to-moderate impact (0–30%) [56–60]. One twin study examined the etiology of IPV victimization, estimating small-to-moderate genetic (15–25%) and very large nonshared environmental influences (75–85%) [61]. Although twin studies of revictimization are scarce, one study found moderate genetic (*r*_*g*_ = 0.22–0.37) and small nonshared environmental correlations (*r*_*e*_ = 0.05–0.16) on the association between maltreatment and subsequent sexual assault victimization [62] – a form of violence often perpetrated by partners [63]. The extent of the genetic and environmental sources of covariation between maltreatment and IPV victimization remain, however, unexplored.

The presence of genetic influences on maltreatment and IPV may seem surprising. However, in a genetically informed design, when a heritable trait is associated with elevated risk for a particular exposure, the exposure itself will exhibit a heritable component. For example, since borderline personality is partly heritable and associated with both maltreatment and IPV victimization [64–66], genetic vulnerability to borderline personality may contribute to the genetic sources of covariation between these two experiences. Long recognized in criminological research [67], the role of personal characteristics in victimization remains understudied, possibly due to concerns that findings may be misrepresented as placing blame on victims [68, 69]. However, successful prevention requires support for those who may be most vulnerable to victimization, alongside measures targeting perpetrators, and comprehensive knowledge of the pathways leading to victimization, including personal vulnerabilities [46, 70].

In this study, we used a genetically informed design to address two research questions. First, to what extent is the association between maltreatment and subsequent IPV victimization attributable to common etiological factors? Based on findings concerning other forms of revictimization [62, 71], we expected moderate-to-large additive genetic and small-to-moderate nonshared environmental influences on this association. Second, does maltreatment exert a potentially causal effect on subsequent IPV victimization, over and above their common etiology? Considering evidence of both causal and common pathways linking these experiences, we predicted a significant but modest causal effect of maltreatment on subsequent IPV victimization. Additionally, based on preliminary evidence of sex differences in the etiology of maltreatment and adult sexual assault victimization [56, 62], we explored possible etiological sex differences in the association between maltreatment and subsequent IPV victimization. We also conducted exploratory analyses to examine the phenotypic and etiological relationships between maltreatment, IPV victimization, and aggression. IPV perpetration often co-occurs with IPV victimization [72], but it is not currently measured in TEDS, preventing direct analysis. Although not an equivalent construct, aggression is moderately associated with IPV perpetration [73] and was included as a proxy to explore its potential role in revictimization.

## Materials and methods

### Participants

We analyzed data from the Twins Early Development Study (TEDS), an ongoing longitudinal data collection involving twins born in England and Wales between 1994 and 1996. The TEDS was approved by the ethics committee at King’s College London. Families were identified using electronic birth records and invited to participate via the Office of National Statistics, with response rates ranging from 84.40% at first contact to 75.60% in emerging adulthood [74]. Parents, teachers, and twins took part at various waves, providing informed consent. Zygosity was determined using DNA marker testing or a parent-reported questionnaire on physical similarity showing 95% accuracy [75]. Ethnicity was representative of the UK population in the 1990s and remained largely stable (92.00% White at first contact, 93.90% at age 26). The proportion of twins whose parents reported holding an advanced secondary degree and being employed at first contact increased slightly (e.g., 43.20% of twins had mothers in employment at first contact, 46.80% at age 26). The proportion of twins holding a university degree was somewhat higher than the national estimate (66.40% at age 26 vs. 56.00%) as was the proportion of those in employment (88.40% vs. 84.00%), although median income was in line with the national figure for age 22–29 [76]. For more details on recruitment procedures and sample representativeness, see [74, 76, 77].

From the original sample with non-missing data on the variables of interest (*N* = 13,306, corresponding to 48.35% of the 27,518 twins enrolled at first contact), we excluded 512 participants due to serious medical and perinatal conditions or missing essential background variables (e.g., zygosity). The final sample for the current study comprised 12,794 participants (6397 twin pairs), including 2224 monozygotic twin pairs (2792 women, 1656 men) and 4173 dizygotic twin pairs, of whom 2048 were same-sex (2496 women, 1600 men) and 2125 were opposite-sex. Participants were predominantly heterosexual (age 21: 79.14, age 26: 84.75%) and in a relationship (age 21: 58.16, age 26: 65.20%). We analyzed sex assigned at birth (58% women, 42% men), acknowledging that 1.49% of TEDS participants (0.95% of women-at-birth, 0.54% of men-at-birth) reported not identifying with it at age 26. Demographic information is reported in Table 1.

**Table 1:** Demographic information.

	Age 21		Age 26	
	*n*	%	*n*	%
Education				
None	0	0.00%	25	0.32%
GCSE	3	0.11%	643	8.11%
A-level	12	0.42%	1162	14.65%
Undergraduate / vocational degree	1939	68.61%	4265	53.78%
Masters degree	795	28.13%	1779	22.43%
PhD	77	2.72%	56	0.71%
Missing	9968		4864	
Employment				
Studying	3310	36.28%	446	5.50%
Working	4830	52.94%	7182	88.64%
Unemployed	883	9.68%	361	4.46%
Looking after home / family	100	1.10%	113	1.39%
Missing	3671		4692	
Relationship status				
Single	3836	41.69%	2807	34.51%
In relationship	3879	42.15%	1639	20.15%
Living with partner	1362	14.80%	3169	38.96%
Married	111	1.21%	495	6.09%
Widowed/separated/divorced	14	0.15%	23	0.28%
Missing	3592		4661	
Sexual orientation				
Heterosexual	7281	79.14%	6691	84.75%
Homosexual	325	3.53%	413	5.23%
Bisexual	1394	15.15%	669	8.47%
Other	200	2.17%	122	1.55%
Missing	3594		4899	

*Note:* Percentages are calculated excluding missing cases for each variable at each time point. Education: At age 21, “Studying” includes participants in apprenticeship or employment training; at age 26, “Unemployed” includes those unable to work due to illness or disability; “Other” includes, at age 21, gap year or traveling and, at age 26, unpaid work. GCSEs = General Certificate of Secondary Education; A-level = Advanced level qualification. Relationship status: “In relationship” includes participants in exclusive and non-exclusive relationships not living with their partner at age 21, and it includes “other” at age 26. Sexual orientation: “Bisexual” includes, at age 21, participants who reported being equally attracted to males and females or being attracted to the opposite sex sometimes; at age 26, it includes those who reported being pansexual or fluid; “Other” includes, at age 21, participants who reported little/no sexual orientation, being unsure, or not knowing, and, at age 26, those who reported being asexual or “other”.

### Measures

We analyzed self-reported continuous measures of maltreatment and IPV victimization collected at ages 21 and 26. See Table 2 for details on items, scoring, and composite scores computation. Maltreatment during childhood was measured retrospectively using the ‘Life at 22 + ’ scale at age 21 [78] and the 5-item form of the Childhood Trauma Screener (CTS) [79] at 26. Both measures assessed emotional abuse (e.g., Life at 22 + : “How often did an adult say hurtful or insulting things to you?”; CTS: “I felt that someone in my family hated me”) and physical abuse (e.g., Life at 22 + : “Did an adult push, grab or shove you?”; CTS: “People in my family hit me so hard that it left me with bruises or marks”). The CTS additionally assessed emotional neglect (“I felt loved”, reverse coded), physical neglect (“There was someone to take me to the doctor if I needed it”, reverse-coded), and sexual abuse (“Someone molested me [sexually])”.

**Table 2:** Questionnaire measures.

Variable	Age	Measure	Items	Response options
CM	21	Life at 22+ questionnaire	When you were a child, how often: 1. Did an adult in your family shout at you? 2. Did an adult say hurtful or insulting things to you? 3. Did an adult push, grab or shove you? 4. Did an adult smack you for discipline? 5. Did an adult punish you in a way that seemed cruel? 6. Did an adult threaten to kick, punch, or hit you with something that could hurt you, or physically attack you in another way? 7. Did an adult actually kick, punch, or hit you with something that could hurt you, or physically attack you in another way? 8. Did an adult hit you so hard it left you with bruises or marks?	Never(0), Rarely (1), Sometimes (2), Often (3), Very often (4)
	26	Shortened version of the Childhood Trauma Screener (CTS)	When I was growing up: 1. I felt loved [reverse-coded] 2. People in my family hit me so hard that it left me with bruises or marks 3. I felt that someone in my family hated me 4. Someone molested me (sexually) 5. There was someone to take me to the doctor if I needed it [reverse-coded]	Never true(0), Rarely true (1), Sometimes true (2), Often true (3), Very often true (4)
IPV	21	CDC Violence Prevention questionnaire	Your partner (current or past): 1. Got very jealous or tried to control your life 2. Tried to keep you away from your family or friends 3. Sometimes said insulting things or threatened you 4. Pushed, hit, kicked, or otherwise physically hurt you 5. You were afraid to disagree with your partner (current or past) because you thought they might hurt you or other family members 6. Made you feel scared or frightened	Strongly disagree (1), Disagree (2), Neither agree nor disagree (3), Agree (4), Strongly agree (5)
	26	Domestic Abuse scale of the Adult Trauma Screener	Since I was sixteen: 1. I have been in a confiding relationship [reverse-coded] 2. A partner or ex-partner deliberately hit me or used violence in any other way 3. A partner or ex-partner repeatedly belittled me to the extent that I felt worthless 4. A partner or ex-partner sexually interfered with me, or forced me to have sex against my wishes	Never true(0), Rarely true (1), Sometimes true (2), Often true (3), Very often true (4)

*CM* childhood maltreatment, *IPV* intimate partner violence. Composite scores of maltreatment and IPV were computed as mean scores, requiring at least half of the items to be non-missing. Composite scores of maltreatment were additionally multiplied by the number of component items. At age 21, maltreatment scores ranged from 0–32, IPV scores ranged from 1–5; at age 26, maltreatment scores ranged from 0–19, IPV scores ranged from 0–4. For more details on the measures, see: Life at 22+ questionnaire [78]; Shortened version of the Childhood Trauma Screener [79]; CDC Violence Prevention questionnaire [80]; Domestic Abuse scale of the Adult Trauma Screener [81].

IPV from current or past partners was measured at age 21 using 6 items from the Centers for Disease Control and Prevention (CDC) Violence Prevention questionnaire [80] and 4 items from the Adult Trauma Screener at 26 (ATS) [81, 82]. Both measures assessed emotional abuse (e.g., CDC questionnaire: “Your partner sometimes said insulting things or threatened you”; ATS: “A partner or ex-partner repeatedly belittled me to the extent that I felt worthless”) and physical abuse (CDC questionnaire: “Your partner pushed, hit, kicked, or otherwise physically hurt you”; ATS: “A partner or ex-partner deliberately hit me or used violence in any other way”). Additionally, the CDC questionnaire assessed controlling and threatening behavior (e.g., “Your partner got very jealous or tried to control your life”) and the ATS assessed relationship insecurity (“I have been in a confiding relationship”, reverse-coded) and sexual abuse (“A partner or ex-partner sexually interfered with me, or forced me to have sex against my wishes”).

Measures were selected by the TEDS team to capture the constructs of interest and for consistency with other large-scale surveys (e.g., UK Biobank). These measures of maltreatment [78, 83] and IPV victimization [81, 84] have shown acceptable psychometric properties in previous studies. In the current study, internal consistency and reliability were excellent at age 21 (maltreatment: Cronbach’s *α* = 0.90, omega total ω_t_ = 0.91, intraclass correlation coefficient ICC = 0.89; IPV victimization: *α* = 0.91, ω_t_ = 0.93, ICC = 0.91) and acceptable at 26 (maltreatment: *α* = 0.65, ω_t_ = 0.72, ICC = 0.67; IPV victimization: *α* = 0.64, ω_t_ = 0.77, ICC = 0.89). Despite some variation in the psychometric properties between measurement occasions, no significant shifts in item distribution were observed (SM1, ST1). The use of different measures at different time points thus served as a conceptual replication.

For the descriptive analyses, we classified participants as exposed to victimization if they scored 1.5 standard deviations above the sample mean on the composite scores of maltreatment and/or IPV, at each time point. This cut-off classified participants as exposed to victimization if they had experienced maltreatment or IPV at least “sometimes” on average (or, for IPV at age 21, those who at least “agreed” to have experienced IPV). At age 21, participants without prior intimate relationships (11.29% of the sample) were included among those reporting no IPV victimization, accounting for 14.40% of this subsample. Information about how many participants had no prior relationships at age 26 was not collected.

### Statistical analyses

Data preparation included assessing the distribution and psychometric properties of our measures, performing multiple imputation, and normalizing variables (SM1). The resulting dataset was used for all except descriptive analyses. We adopted a conservative significance level of *p* < 0.001 and, following recent recommendations [85], we considered correlation and regression coefficients as very small when ≦ 0.10, small when 0.11–0.20, moderate when 0.21–0.30, large when 0.31–0.40, and very large when ≧ 0.40. We followed the conventions in behavioral genetics research for interpreting the magnitude of the genetic and environmental components from twin models, considering their relative contribution to the total variance [86]. Heritability estimates ≦ 0.20 are generally considered modest, while those ≧ 0.60 are typically considered large (e.g., [87, 88]). Definite boundaries for moderate estimates have not been formally set, to our knowledge, but estimates falling in between these two boundaries are typically described as moderate (e.g., [60, 89]). We compared nested models using the likelihood ratio test [90].

We conducted descriptive analyses by inspecting how many participants exceeded our cut-offs for maltreatment and IPV victimization. Next, we compared the proportion of participants reporting IPV victimization among those exposed vs. not to maltreatment. We then estimated phenotypic regressions accounting for the non-independence of observations using generalized estimating equation (GEE) [91]. We tested whether the effect of maltreatment on IPV victimization differed by sex by including sex as an interaction term and inspecting whether the term was significant.

Using bivariate correlated factors twin models [38], we estimated the proportion of variance in and covariance between maltreatment and IPV victimization due to additive genetic influences (denoted as “A”), shared environmental influences (“C”), and nonshared environmental influences, encompassing measurement error (“E”), and the extent of the overlap in their etiological influences. Using bivariate sex limitation models [92, 93], we explored possible sex differences in the etiology of the association between maltreatment and IPV (see SM2). Using direction-of-causation twin models [94, 95], we tested the presence of a causal link from maltreatment to IPV victimization (see SM3). Twin analyses were conducted in R using packages OpenMx [96] and umx [97]. Details of the exploratory analyses of aggression are provided in SM4.

## Results

Descriptive results are reported in Table 3. About 7% of participants reported experiencing maltreatment, about 8% reported IPV victimization. Slightly more women than men reported maltreatment; about twice as many women than men reported IPV. Participants who reported experiencing maltreatment were three times more likely than their peers to report IPV at age 21, and four times more likely at 26. Risk of IPV following maltreatment was comparable across sexes, but slightly higher in men.

**Table 3:** Prevalence rates.

	Age	Full sample					Women					Men				
		*n (N)*	%	CM	No CM	RR	*n (N)*	%	CM	No CM	RR	*n (N)*	%	CM	No CM	RR
CM	21	621 (8385)	7.41%	–	–	–	447 (5322)	8.40%	–	–	–	174 (3063)	5.68%	–	–	–
	26	569 (7708)	7.38%	–	–	–	435 (5058)	8.60%	–	–	–	134 (2650)	5.06%	–	–	–
IPV	21	772 (8980)	8.60%	18.84%	6.54%	2.88	592 (5647)	10.48%	21.74%	8.90%	2.44	180 (3333)	5.40%	18.49%	4.44%	4.17
	26	590 (7687)	7.68%	26.19%	6.18%	4.24	469 (5036)	9.31%	28.77%	7.49%	3.84	121 (2642)	4.58%	18.80%	3.83%	4.91

*CM* childhood maltreatment, *IPV* intimate partner violence, *N* indicates the number of participants with non-missing values on the corresponding composite score, *No CM* Participants who reported no maltreatment history at the corresponding time point, *RR* Risk Ratio.

Regression models revealed significant moderate positive associations between maltreatment and IPV (age 21: *β* = 0.25 [95% CIs = 0.23, 0.27], *p* < 0.001; age 26: *β* = 0.30 [0.28, 0.33], *p* < 0.001). Although larger for women, regression coefficients did not differ significantly by sex (age 21: *β*_women_ = 0.26 [0.24, 0.29], *β*_men_ = 0.21 [0.17, 0.25], *p* = 0.011; age 26: *β*_women_ = 0.31 [0.28, 0.34], *β*_men_ = 0.28 [0.23, 0.34], *p* = 0.373).

**Fig. 2:**
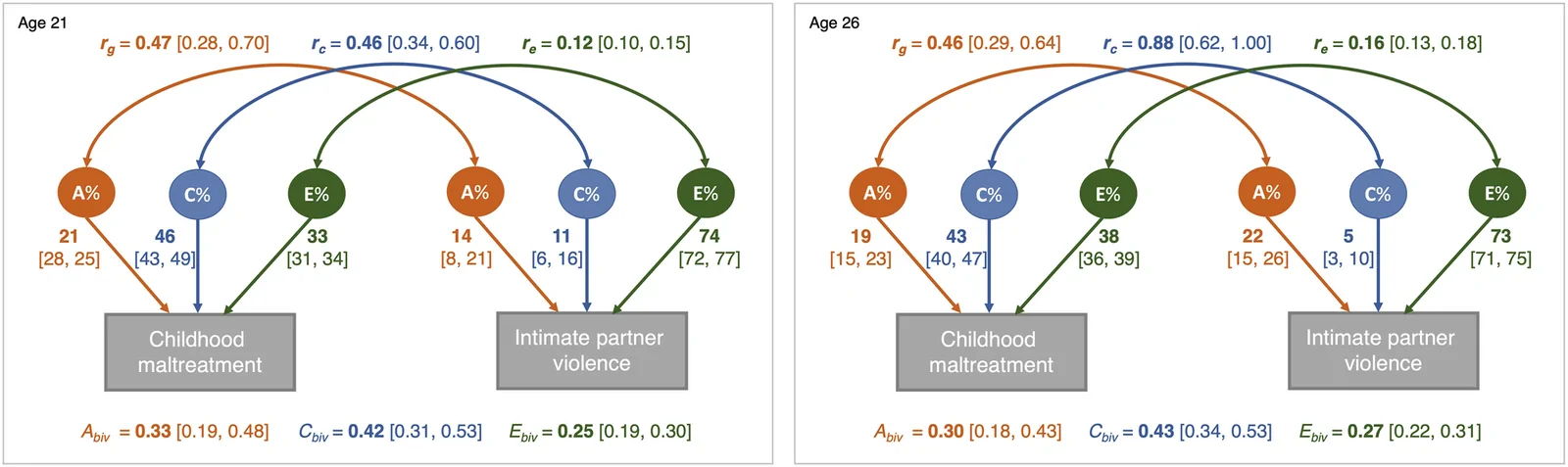
Bivariate correlated factors twin models of childhood maltreatment and intimate partner violence victimization. r_g_ = genetic correlations, r_c_ = shared environmental correlations, r_e_ = nonshared environmental correlations; A% = Additive genetic influences; C% = Shared environmental influences; E% = Nonshared environmental influences; A_biv_ = bivariate heritability; C_biv_ = bivariate shared environmental influences; E_biv_ = bivariate nonshared environmental influences. All estimates are statistically significant (*p* < 0.001); confidence intervals are reported in brackets.

Bivariate correlated factors twin models (Fig. 2) indicated large shared environmental, moderate nonshared environmental, and small-to-moderate genetic influences on maltreatment. Nonshared environmental influences accounted for a very large proportion of the variance in IPV, genetic influences were small-to-moderate, shared environmental influences were small. The association between maltreatment and IPV was attributable to large shared environmental, moderate genetic, and small nonshared environmental influences. We also found very large shared environmental and genetic correlations and small nonshared environmental correlations between maltreatment and IPV.

Sex limitation analyses (ST4) indicated quantitative sex differences in the environmental variance components at both time points. Specifically, equating shared and nonshared environmental components between women and men significantly worsened model fit (*p* < 0001), whereas equating the genetic component did not (*p* = 1.000). Inspecting estimates revealed minor sex differences in the etiology of maltreatment, including larger shared environmental influences in men than women at both time points (age 21: 0.75, [0.71, 0.79] vs. 0.59 [0.55, 0.62], *p* < 0.001; age 26: 0.67 [0.64, 0.73] vs. 0.53 [0.49, 0.57], *p* < 0.001) and larger nonshared environmental influences in women than men at age 21 (0.39 [0.36, 0.42] vs. 0.31 [0.29, 0.33], *p* < 0.001). Qualitative sex limitation models failed to converge at both time points, precluding definitive conclusions on qualitative sex differences in our sample.

**Fig. 3:**
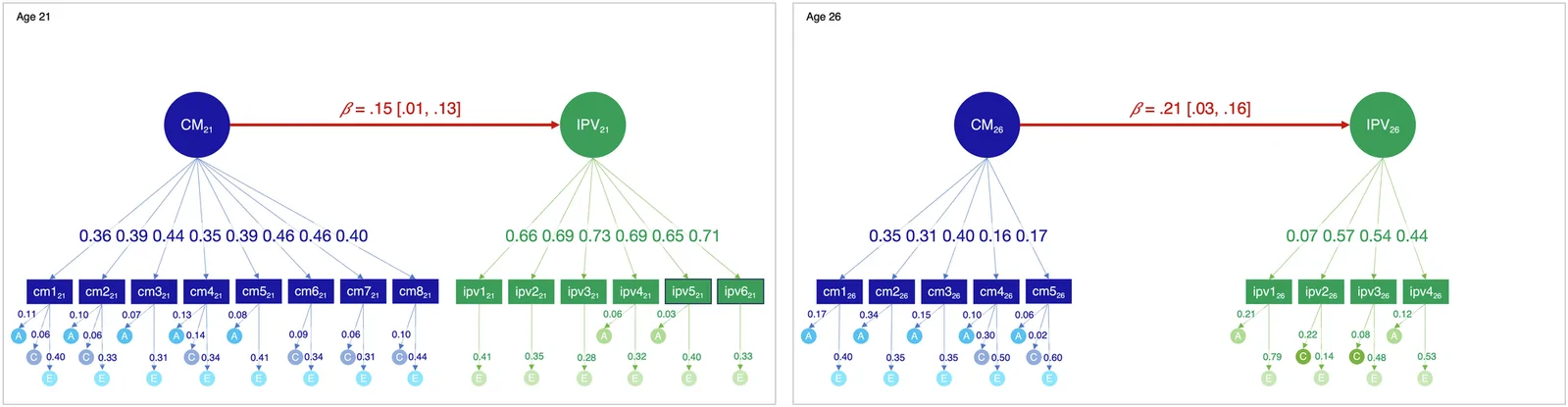
Direction-of-causation model illustrating the causal impact of childhood maltreatment (CM) on intimate partner violence (IPV) victimization. Nonsignificant paths and confidence intervals are omitted for ease of visualization. Direction of causation models replace one or more of the variance components for the common factors with regression paths directly from one phenotype to the other; therefore, factor components are not displayed.

The direction-of-causation model stipulating a causal effect of maltreatment on IPV showed a comparable fit to the baseline noncausal model across time points and was therefore retained (model comparisons in ST5). The estimated effect of maltreatment on IPV was significant and positive, small at age 21 and moderate at 26 (Fig. 3). Alternative models testing a direction of causal effect from IPV to maltreatment could predictably be discarded, as IPV occurred after maltreatment. These models showed decrease in fit compared to the baseline; with the exception of the reciprocal model that showed comparable fit, but with the effect of IPV on maltreatment being negative and marginal at age 21 and nonsignificant at 26, suggesting a failure to accurately represent the data.

Exploratory analyses (SM4) revealed higher levels of aggression among participants exposed to maltreatment, IPV victimization, or both, compared to those without such exposures, across sexes (ST2). However, aggression did not moderate the association between maltreatment and IPV victimization. Both experiences showed large genetic, very large shared environmental, and small non-shared environmental correlations with aggression (ST3).

## Discussion

Although individuals with a history of childhood maltreatment are particularly vulnerable to IPV victimization, research has made little progress in elucidating the mechanisms driving this form of revictimization. We addressed this gap using a quasi-experimental genetically informed design to discern causal and common pathways linking maltreatment to subsequent IPV victimization. Results indicated that environmental factors shared by co-twins, as well as genetic factors, mostly explain the association between maltreatment and subsequent IPV victimization. Furthermore, results supported the presence of a possible causal effect of maltreatment on subsequent IPV victimization, which persisted even when adjusting for common etiological influences.

### The common risk pathways to childhood maltreatment and intimate partner violence are mostly shared environmental and genetic in origin

Shared environmental factors were particularly influential in explaining individual differences in exposure to both maltreatment and IPV victimization. Shared environmental influences on maltreatment have been previously documented [56]. Since maltreatment is typically perpetrated by parents, it seems intuitive that its origins may lie primarily in environmental factors shared by co-twins raised together. However, negative parental behavior often varies between siblings, also as a result of genetically-influenced child traits [98, 99]. Therefore, our finding that maltreatment affected participants similarly regardless of their genetic similarity implies that abusive or neglectful parent behavior may be shaped more strongly by the parent’s characteristics than by their particular relationship with each child, in line with prior research [100].

Shared environmental influences on the association between maltreatment and IPV victimization align with criminological findings on lifestyle and routine activities increasing revictimization risk [67, 101]. Activities like occupational demands in high-risk environments and housing instability are intergenerationally correlated and can limit access to protective resources, potentially discouraging maltreatment survivors from reporting subsequent victimization and making them vulnerable targets for perpetrators [102–104]. While the impact of such structural risk factors at a group level is known, our results highlight their potential role in driving individual differences too. Moreover, by suggesting that stressors inherent in adverse family environments may not only increase the risk of perpetrating violence but also of becoming a victim, our findings lend support to applying the general strain theory of violence to the study of victimization [105, 106].

Additionally, shared environmental correlations increased from age 21 to 26, which may reflect a longitudinal increase in the impact of shared influences, such as cultural norms, that persist when co-twins grow up [107, 108]. These mechanisms may contribute to the observed escalation in IPV risk among survivors of maltreatment from age 21 to 26, although we cannot rule out that measurement discrepancies between these time points may also partly account for this finding. While differences between the measures used at ages 21 and 26 precluded direct comparisons, the finding of a stronger association between maltreatment and IPV victimization when these experiences were reported at age 26 was observed across various methods (risk ratios, phenotypic regression, direction of causation models) and despite lower psychometric properties of the measures used at age 26, which would be expected to attenuate associations. Therefore, results suggest that risk of revictimization may accumulate over time. This is consistent with evidence that survivors of maltreatment often experience difficulties that gradually restrict the range of available behavioral responses and lead to ‘thinner’ social networks, increasing vulnerability to unsafe relationships [11, 36, 37]. Conversely, the modest nonshared environmental influences on the association between maltreatment and IPV victimization imply that unique exposures – including distinct intimate partners – are less central to this revictimization pattern. These findings thus complement existing IPV theories focusing predominantly on perpetrator characteristics, by underscoring the importance of the victim’s background in the pathways to IPV risk [46].

The second main source of covariation between maltreatment and IPV victimization was genetics. Since genes cannot affect victimization directly, genetic estimates likely reflect genetic propensities that increase susceptibility to experiencing or reporting victimization. The large genetic correlations observed between maltreatment, IPV victimization, and aggression in our exploratory analyses point to aggression as one possible trait contributing to the heritable component of revictimization. Other externalizing traits that have been associated with revictimization include low self-control [39, 109] and high risk-taking [110, 111]. In the absence of protective factors, genetic susceptibility for these traits may contribute to narrowing socialization opportunities over time. For example, children with a genetic predisposition toward aggression may elicit harsher parenting [112] and, later in life, may choose partners who display aggressive behaviors, consistent with the frequent bidirectionality of IPV [113]. Beyond externalizing traits, heritable differences in personality may contribute to differences in victimization risk and experiences [114]. For example, preliminary evidence suggests that individuals displaying exploitative behaviors tend to pursue partners with personality traits they view as manipulable, such as high emotionality and agreeableness [115, 116]. Additionally, the genetic component of revictimization may encompass genetic influences on psychological processes, including cognitive biases, affecting how individuals perceive, interpret, and recall their experiences [117–119]. Future research should clarify how heritable traits and psychological processes influence the selection and attraction of intimate partners following maltreatment. The intergenerational transmission of violence, including the association between maltreatment and subsequent IPV victimization, has been usually attributed to social learning processes, where maltreated individuals may adopt beliefs that normalize violence, thereby learning to tolerate or use it [120, 121]. However, it is clear from prior studies, typically reporting small effect sizes, that the risk factors for revictimization are complex and that multiple methodological approaches are required to address this complexity [122, 123]. Our findings make an important contribution to this effort by underscoring the importance of considering genetic vulnerabilities, alongside environmental ones, when studying the intergenerational transmission of violence.

Since genetic risk operates probabilistically and in combination with the environment, it can be mitigated by preventative measures. Alongside examining measures that account for individual-level vulnerabilities, future research should differentiate between pre-existing genetic effects, potentially exacerbating the impact of maltreatment (e.g., genetic risk for low self-control reducing resilience to trauma), from novel genetic effects exacerbated by maltreatment. This research can inform policy decisions about the prioritization of interventions aimed at reducing the risk of IPV among individuals exposed to maltreatment [8]. For instance, identifying specific pathways linking maltreatment to unsafe relationships, over and above their common risk factors (e.g., socioeconomic disadvantage), may illuminate targets for secondary interventions aimed at preventing IPV in this group. Identifying risk pathways for young adults seems particularly crucial because early intimate relationships may underpin future family dynamics, making this research vital for informing interventions to disrupt the intergenerational transmission of violence [68, 124].

### The causal effect of childhood maltreatment on intimate partner violence persists beyond their common risk pathways

Even when accounting for all possible common risk factors, maltreatment showed a small-to-moderate effect on subsequent IPV victimization. Our findings thus offer initial evidence of a potential causal effect of maltreatment in exacerbating processes that may lead survivors into unsafe intimate relationships. Phenotypic studies have typically estimated a moderate-to-large effect when accounting for measured covariates. For instance, a cross-sectional study using structural equation modeling estimated a large effect of self-reported maltreatment on IPV victimization when adjusting for socioeconomic indicators known to confer risk for both experiences (*β* = 0.33) [48]. A prospective study using multivariable logistic regression estimated a moderate effect of substantiated maltreatment on IPV victimization when adjusting for individual-level risk factors (e.g., aggressive behavior) and family-level risk factors (e.g., social deprivation) (adjusted/unadjusted odds ratio = 2.12/3.19); [49]. Our results demonstrate that the moderate effects identified using phenotypic methods, including our own regression analyses, may be inflated due to unmeasured sources of confounding. Genetically informed studies can thus guide future research into how risk for revictimization operates.

Although the precise causal pathways from maltreatment to IPV victimization have yet to be determined, various mediators have been identified, showing small indirect effect sizes. Examples include post-traumatic stress disorder (*β* = 0.18–0.19 [125, 126]) and emotional dysregulation (*β* = 0.06–0.17 [34, 35]). Maltreatment is also associated with difficulties in socio-cognitive processes including sense of agency and ability to discern trustworthiness [36, 127, 128], that are known to influence relationship dynamics [37, 129, 130]. Future studies should examine how psychological processes impacted by maltreatment elevate risk of harmful intimate relationships. They should also assess the effect of interventions like trauma-focused cognitive-behavioral therapy on interpersonal outcomes, including the development of safe intimate relationships following maltreatment [131, 132]. Progress in this research is indispensable for enhancing prevention efforts and interrupting cycles of revictimization.

### Strengths and limitations

This study is the first to apply a quasi-experimental genetically informed approach to study what drives the association between maltreatment and subsequent IPV victimization. By accounting for unmeasured sources of confounding, this approach strengthened our ability to draw causal inferences [52, 133], providing robust evidence of both causal and common risk pathways from maltreatment to subsequent IPV victimization. This study also benefits from the largest sample size on this research question to date (*N* = 12,794), increasing statistical power and confidence in the results. The compelling findings open avenues for further research aimed at illuminating intervention targets to prevent revictimization.

Some limitations also warrant consideration. First, the TEDS dataset lacks some comprehensive measures relevant to our research questions. For instance, due to the limited number of items per type of maltreatment and IPV, we were unable to investigate whether phenotypic and etiological associations differ across specific types of maltreatment and IPV. However, since poly-victimization is regrettably the norm – i.e., different forms of victimization frequently co-occur [134, 135] – and different types of maltreatment all increase risk of IPV victimization [12], an unspecific approach remains appropriate. Additionally, we were unable to examine the distinctions between victims, perpetrators, and victim-perpetrators of IPV due to the absence of IPV perpetration measures in TEDS. To partially address this gap, we explored the role of aggression, a trait known to be moderately associated with IPV perpetration [73]. Many questions also remain about the extent and etiology of the intergenerational transmission of violence, which should be explored in future studies using genetically sensitive designs in datasets that include relevant measures collected across generations, which were unavailable here. Second, we used retrospective self-reports of victimization. While such measures may yield inflated prevalence, the converse is true when relying on prospective, official records of victimization [136]. Moreover, self-report measures capture subjective perceptions of victimization that are most strongly associated with mental health outcomes [137, 138] and, possibly, vulnerability to IPV. Third, our sample lacked diversity, primarily comprising White heterosexual participants. Future research could investigate potential differences in etiological pathways to revictimization among marginalized groups, including ethnic minorities and LGBTQ+ individuals [139, 140]. Lastly, since some assumptions of the direction-of-causation model may be violated, such as no mutual effects between co-twins [141], replication with alternative statistical approaches for causal inferences is warranted.

In conclusion, this study sheds light on the nature of the longitudinal association between maltreatment and IPV victimization. Our findings corroborate previous evidence that individuals exposed to maltreatment face a considerably increased risk of IPV, and further highlight an escalation in this risk over time. Genetically informed analyses revealed the substantial contribution of shared environmental and genetic factors common to maltreatment and IPV in driving their association. However, maltreatment remained causally related to IPV victimization even when accounting for their common etiology, albeit with a modest effect size. These findings not only offer novel theoretical insights into the processes underlying revictimization but also hold implications for policy and intervention strategies. In particular, they underscore the need for early interventions to mitigate the impact of maltreatment on intimate relationships, and the importance of tailored approaches to identification and management of IPV risk among maltreatment survivors, including measures tailored to personal and familial vulnerabilities.

## Supplementary information

Supplemental materials

## Data Availability

This study is part of a pre-registered project (osf.io/byqm4). Code is publicly accessible (osf.io/4rv3x). TEDS data are available upon request (teds.ac.uk/researchers/teds-data-access-policy).
