# Supplementary material for: Causal and common risk pathways linking childhood maltreatment to later intimate partner violence victimization
Source: Mol Psychiatry. 2024 Nov 2;30(5):2027–37. doi: 10.1038/s41380-024-02813-0 (PMC12015119; doi:10.1038/s41380-024-02813-0)
Supplement: Supplementary file 1 — Supplemental materials [file 41380_2024_2813_MOESM1_ESM.docx]

**Causal and common risk pathways linking childhood maltreatment to later intimate partner violence victimization**

# Supplementary materials (SM)

## SM1. Data preparation

Data preparation was conducted in R (1) using the packages dplyr (2), psych (3), naniar (4), and mice (5). First, we estimated the psychometric properties of our composite scores of maltreatment and IPV victimization (Table ST1). Across measurement occasions, composite scores demonstrated good internal consistency, despite a decline from age 21 to age 26, possibly attributable to the use of less concrete and specific items at age 26 (Table 2). Factor analysis supported the presence of a single factor underlying the items that comprised each measure, but testing measurement invariance was precluded due to the use of different items at age 21 and 26. Next, we inspected the pattern of missing data and found that 9,018 participants had a missing value on one to three of the four composite scores. Little’s Missing Completely At Random (6) test yielded significant results, χ^2^ (494) = 13344.1, *p* < .001, meaning that the pattern of missingness was not completely random. More data points were missing for variables collected at age 26 vs. 21, consistent with participant dropout at subsequent waves. Multiple imputation was conducted using the Multiple Imputation by Chained Equations Bayesian method with 30 iterations (i.e., missing values imputed repeatedly to create 30 complete datasets, then combined into a pooled dataset using Rubin’s rules (7)). The imputation converged adequately, as indicated by stable summary statistics (e.g., mean values for the imputed data on average .03 smaller than mean values for the original data). Skewness and kurtosis were within the normal range (-3–3 and -10–10, per structural equation modelling guidelines (8)). Lastly, we standardized variables for analysis by centering them to have a mean of 0 and scaling them to have a standard deviation of 1, and we examined their distribution across measurement occasions. Longitudinal within-construct correlations were very large, indicating good stability, but not perfect, which was expected due to the use of different measures of the same constructs at ages 21 and 26 (maltreatment: *r* = 0.73 [95%CI: 0.70, 0.73]; IPV victimization: *r* = 0.51 [0.49, 0.53]). We further investigated any changes in the distribution using the Wilcoxon signed-rank test for clustered data (9) which indicated no significant shifts from age 21 to 26 in maltreatment scores (*p* = 0.511) or in IPV scores (*p* = 0.542).

## SM2. Specification of sex limitation models

We explored possible etiological sex differences in maltreatment and IPV using bivariate sex limitation twin models (10,11). Quantitative sex differences are indexed by differences in the magnitude of the ACE variance components common to women and men, and indicate that the impact of the same etiological factors influencing both sexes is stronger on one sex relative to the other. Qualitative sex differences are indexed by differences in the etiological correlations between opposite-sex twins, and suggest the presence of distinct etiological influences in the two sexes.

At each time point, we first fitted a model accommodating both quantitative and qualitative sex differences. Quantitative sex differences were modeled by allowing distinct A, C, and E variance components in males and females; qualitative sex differences were modeled by allowing distinct between-variable genetic or shared environmental correlations in opposite-sex DZ twins (due to limitations on the degrees of freedom allowed by this model, genetic and shared environmental correlations could not be freed simultaneously). Next, we tested for qualitative sex differences by sequentially equating the etiological correlations (*r_g_*, *r_c_*, and *r_e_*) between opposite-sex twins. We then tested for quantitative sex differences, by sequentially equating the A, C, and E variance components between males and females, in addition to the etiological correlations.

## SM3. Specification of direction-of-causation models

We used direction-of-causation twin models (12,13) to investigate the presence of a causal link from maltreatment to IPV. Direction-of-causation models test hypotheses about causation between variables measured cross-sectionally in genetically related individuals, by treating the variance components of one trait as instrumental variables in the prediction of a second trait (14). To reduce the impact of measurement error and increases power for hypothesis testing (15), we estimated two latent factors of maltreatment and IPV, each capturing the shared variance between their individual items. We then specified a baseline non-causal model, equivalent to a bivariate Cholesky decomposition (16), and a reciprocal model allowing direct regression paths between the two latent factors. Next, we specified two unidirectional causation models, by constraining the etiological influences on IPV to be fully mediated by the influences on maltreatment, and vice versa. Given the temporal ordering of our variables, we considered the path from IPV to maltreatment as a “sanity check” to test reporting bias (i.e., IPV causing individuals to report maltreatment). Lastly, we compared reciprocal and unidirectional causal models against the baseline.

## SM4. Exploratory analyses: Phenotypic and etiological relationships between maltreatment, intimate partner violence, and aggression at age 21

**Methods:** In addition to the main analyses, we conducted exploratory analyses to investigate the phenotypic and etiological relationship between maltreatment, IPV, and aggression, as an indicator of possible IPV perpetration proclivity. Aggression was measured at age 21 using the eight-item Brief Aggression Questionnaire (BAQ) (17). The BAQ includes three items assessing physical aggression (e.g., “Given enough provocation, I may hit another person”), three items assessing verbal aggression (e.g., “I tell my friends openly when I disagree with them”), and two items assessing anger and hostility (e.g., “I have trouble controlling my temper”). A composite score was computed as mean of all BAQ items, with at least half of the items required to be non-missing. This composite score showed good internal consistency (Cronbach’s 𝛼 = 0.80). For descriptive analyses, we classified participants as displaying elevated levels of aggression if they scored 1.5 standard deviations above the sample mean on this composite score, consistent with the main descriptive analyses. We used phenotypic regression with generalized estimating equation (GEE) to investigate whether aggression moderated the relationship between maltreatment and IPV at age 21, and we estimated multivariate correlated factors twin models to determine the sources of covariance and the etiological correlations between each type of victimization (maltreatment and IPV) and aggression at age 21.

**Results:** Exploratory results are presented in [Supplementary Tables ST2 and ST3](#_Supplementary_Table_ST2._1).At the phenotypic level, over 7% of the sample displayed elevated levels of aggression; this proportion increased approximately threefold among victims of maltreatment or IPV only, fourfold among victims of both maltreatment and IPV. Although more men than women displayed elevated levels of aggression, maltreatment and IPV increased risk of displaying elevated aggression comparably for women and men. Aggression did not significantly moderate the association between maltreatment and IPV in phenotypic regression analyses (*β* = 0.01 [-0.03, 0.04], *p* = 0.741), regardless of sex (three-way interaction: *β* = 0.03 [-0.04, 0.09], *p* = 0.390). At the etiological level, twin analyses indicated that the association between maltreatment and aggression was moderate and attributable to large genetic and moderate shared and nonshared environmental influences. The association between IPV and aggression was small and similarly attributable to large genetic and moderate shared and nonshared environmental influences. We also found large shared environmental, moderate genetic, and small nonshared environmental correlations between each type of victimization and aggression.

## Supplementary Tables (ST)

#### Supplementary Table ST1. Internal consistency, reliability, and construct validity of the composite scores

|  |  | 𝛼 | ω_t_ | *ICC* | χ^2^ | *df* | *p* |
| --- | --- | --- | --- | --- | --- | --- | --- |
| CM | Age 21 | 0.90 | 0.91 | 0.89 | 4581.07 | 20 | < 0.001 |
|  | Age 26 | 0.65 | 0.72 | 0.67 | 273.28 | 5 | < 0.001 |
| IPV | Age 21 | 0.91 | 0.93 | 0.91 | 3745.23 | 9 | < 0.001 |
|  | Age 26 | 0.64 | 0.77 | 0.89 | 62.15 | 2 | < 0.001 |

*Note*. CM = Childhood maltreatment; IPV = Intimate partner violence; 𝛼 = Standardized Cronbach’s alpha; ω_t_ = Omega total, computed as the amount of reliable variance in the observed variables accounted for by a single factor (18); ICC = Intraclass correlation coefficients computed as per the two-way random effects model with single measures approach (19) *χ*^2^ = chi-square test statistic for the Confirmatory Factor Analysis (CFA) models extracting one factor from the selected items, estimated accounting for non-independence of observations using the lavaan package (20); *df* = degrees of freedom (number of parameters estimated in each model), *p* = *p*-value associated with the chi-square test comparing each CFA model with corresponding baseline models representing the null hypothesis that items are uncorrelated.

#### Supplementary Table ST2. Distribution of aggression scores at age 21

|  | Sex | Low AGG (*n*) | High AGG (*n*) | High AGG (%) | Risk Ratio |
| --- | --- | --- | --- | --- | --- |
| Full sample |  | 8277 | 681 | 7.60% |  |
| By sex |  |  |  |  |  |
|  | Female | 5161 | 387 | 6.98% |  |
|  | Male | 3116 | 294 | 8.62% |  |
| By victimization | |  |  |  |  |
| CM only | | 338 | 78 | 18.75% | 3.39 |
| IPV only | | 406 | 69 | 14.53% | 2.63 |
| Both CM and IPV |  | 82 | 26 | 24.07% | 4.35 |
| No CM or IPV |  | 5772 | 338 | 5.53% |  |
| By victimization and sex | |  |  |  |  |
| CM only | Female | 253 | 49 | 16.23% | 3.40 |
|  | Male | 85 | 29 | 25.44% | 3.74 |
| IPV only | Female | 322 | 48 | 12.97% | 2.72 |
|  | Male | 84 | 21 | 20.00% | 2.94 |
| Both CM and IPV | Female | 64 | 19 | 22.89% | 4.79 |
|  | Male | 18 | 7 | 28.00% | 4.11 |
| No CM or IPV | Female | 3650 | 183 | 4.77% |  |
|  | Male | 2122 | 155 | 6.81% |  |

*Note*. CM = Childhood maltreatment; IPV = Intimate partner violence; AGG = Aggression; CM only = Participants reporting maltreatment but not IPV; IPV only = Participants reporting IPV but not maltreatment; Both CM and IPV = Participants reporting both CM and IPV; No CM or IPV = Participants reporting neither maltreatment nor IPV. All measures are assessed at age 21.

#### Supplementary Table ST3. Multivariate correlated factors twin model of maltreatment, IPV, and aggression at age 21

|  | Phenotypic  correlations | | | Covariation  components | | | | | | | | | Etiological  correlations | | | | | | | | |
| --- | --- | --- | --- | --- | --- | --- | --- | --- | --- | --- | --- | --- | --- | --- | --- | --- | --- | --- | --- | --- | --- |
|  | *r* | lCI | uCI | A | lCI | uCI | C | lCI | uCI | E | lCI | uCI | *r_g_* | lCI | uCI | *r_c_* | lCI | uCI | *r_e_* | lCI | uCI |
| CM-AGG | 0.21 | 0.20 | 0.22 | 0.54 | 0.37 | 0.71 | 0.26 | 0.13 | 0.39 | 0.21 | 0.14 | 0.27 | 0.38 | 0.26 | 0.50 | 0.65 | 0.34 | 0.99 | 0.10 | 0.07 | 0.13 |
| IPV-AGG | 0.18 | 0.16 | 0.19 | 0.46 | 0.24 | 0.67 | 0.23 | 0.10 | 0.39 | 0.31 | 0.24 | 0.40 | 0.34 | 0.20 | 0.51 | 0.97 | 0.74 | 1.00 | 0.08 | 0.06 | 0.11 |

*Note*. CM = Childhood maltreatment; AGG = Aggression; IPV = Intimate partner violence; A = additive genetic influences; C = shared environmental influences; E = nonshared environmental influences; lCI = 95% confidence intervals, lower bound; uCI = 95% confidence intervals, upper bound; *r_g_ =* genetic correlation*; r_c_ =* shared environmental correlation; *r_e_* = nonshared environmental correlation. All measures are assessed at age 21.

#### Supplementary Table ST4. Sex limitation models, summary of model comparisons

| Age | Model | EP | Δ fit | Δ *df* | *p* | AIC | Δ AIC |
| --- | --- | --- | --- | --- | --- | --- | --- |
| 21 | Baseline for qualitative sex limitation | 27 |  |  |  |  |  |
|  | Equated *r_g_* | 25 | -22.144 | 2 | 1.000 | 137342.4 | -26.144 |
|  | Equated *r_g_*, *r_c_* | 23 | -13.219 | 4 | 1.000 | 137347.4 | -21.219 |
|  | Equated *r_g_*, *r_c_*, *r_e_* | 22 | -12.552 | 5 | 1.000 | 137346 | -22.552 |
|  | Baseline for quantitative sex limitation | 19 |  |  |  | 137340 |  |
|  | Equated A | 17 | 9.202 | 2 | 0.01 | 137345.2 | 5.202 |
|  | Equated AC | 15 | 90.696 | 4 | < 0.001 | 137422.7 | 82.696 |
|  | Equated ACE | 13 | 129.567 | 6 | < 0.001 | 137457.6 | 117.567 |
| 26 | Baseline for qualitative sex limitation | 27 |  |  |  |  |  |
|  | Equated *r_g_* | 25 | -0.624 | 2 | 1.000 | 137636.4 | -4.624 |
|  | Equated *r_g_*, *r_c_* | 23 | 45.903 | 4 | <0.001 | 137679 | 37.903 |
|  | Equated *r_g_*, *r_c_*, *r_e_* | 22 | 54.23 | 5 | <0.001 | 137685.3 | 44.23 |
|  | Baseline for quantitative sex limitation | 19 |  |  |  |  |  |
|  | Equated A | 17 | 10.85 | 2 | 0.004 | 137686.1 | 6.85 |
|  | Equated AC | 15 | 106.282 | 4 | < 0.001 | 137777.6 | 98.282 |
|  | Equated ACE | 13 | 120.121 | 6 | < 0.001 | 137787.4 | 108.121 |

*Note*. *r_g_ =* genetic correlation*; r_c_ =* shared environmental correlation; *r_e_* = nonshared environmental correlation; A = additive genetic influences; C = shared environmental influences; E = nonshared environmental influences; EP = Estimated parameters; *df* = degrees of freedom; *p* = *p*-value for the model comparison, AIC = Akaike Information Criterion. Model comparisons indicated qualitatively distinct shared and nonshared environmental correlations between women and men at age 26; however, due to non-convergence, these results could be unstable, precluding definitive conclusions. At both age 21 and 26, model comparisons indicated quantitative sex differences, which were due to small etiological differences in maltreatment.

#### Supplementary Table ST5. Direction of causation models, summary of model comparisons

| Model | Age | EP | Δ Fit | Δ *df* | *p* | AIC | Δ AIC |
| --- | --- | --- | --- | --- | --- | --- | --- |
| Baseline | 21 | 77 |  |  |  | 381392.10 |  |
| CM to IPV |  | 75 | 5.69 | 2 | 0.058 | 381393.80 | 1.69 |
| IPV to CM |  | 75 | 129.01 | 2 | < 0.001 | 381517.10 | 125.01 |
| Reciprocal |  | 76 | 0.11 | 1 | 0.740 | 381390.20 | -1.89 |
| Baseline | 26 | 52 |  |  |  | 171293.80 |  |
| CM to IPV |  | 50 | 2.60 | 2 | 0.272 | 171292.40 | -1.40 |
| IPV to CM |  | 50 | 12.23 | 2 | 0.002 | 171302.00 | 8.23 |
| Reciprocal |  | 51 | 0.68 | 1 | 0.409 | 171292.40 | -1.32 |

Note: CM = childhood maltreatment; IPV = intimate partner violence; EP = Estimated parameters; *df* = degrees of freedom; *p* = *p*-value for the model comparison, AIC = Akaike Information Criterion.

**References**

1. R Core Team. R: A language and environment for statistical computing. R Foundation for Statistical Computing, Vienna, Austria; 2022.

2. Wickham H, François R, Henry L, Müller K. Vaughan, D. dplyr: A grammar of data manipulation. R package version. 2023;1(0).

3. Revelle W. psych: Procedures for psychological, psychometric, and personality research. Evanston, Illinois; 2022.

4. Tierney NJ, Cook DH. Expanding tidy data principles to facilitate missing data exploration, visualization and assessment of imputations. arXiv preprint arXiv:180902264. 2018;

5. Van Buuren S, Groothuis-Oudshoorn K. mice: Multivariate imputation by chained equations in R. J Stat Softw. 2011;45:1–67.

6. Li C. Little’s test of missing completely at random. Stata J. 2013;13(4):795–809.

7. White IR, Royston P, Wood AM. Multiple imputation using chained equations: issues and guidance for practice. Stat Med. 2011;30(4):377–99.

8. Kline RB. Principles and practice of structural equation modeling. Guilford publications; 2023.

9. Rosner B, Glynn RJ, Lee MLT. The Wilcoxon signed rank test for paired comparisons of clustered data. Biometrics. 2006;62(1):185–92.

10. Neale MC, Røysamb E, Jacobson K. Multivariate genetic analysis of sex limitation and G× E interaction. Twin Research and Human Genetics. 2006;9(4):481–9.

11. Medland SE. Alternate parameterization for scalar and non-scalar sex-limitation models in Mx. Twin Research and Human Genetics. 2004;7(3):299–305.

12. Gillespie NA, Martin NG. Direction of causation models. Encyclopedia of statistics in behavioral science. 2005;

13. Duffy DL, Martin NG. Inferring the direction of causation in cross‐sectional twin data: Theoretical and empirical considerations. Genet Epidemiol. 1994;11(6):483–502.

14. McAdams TA, Rijsdijk F V., Zavos HMS, Pingault JB. Twins and causal inference: leveraging nature’s experiment. Cold Spring Harb Perspect Med. 2021;11(6):1–22.

15. Heath AC, Kessler RC, Neale MC, Hewitt JK, Eaves LJ, Kendler KS. Testing hypotheses about direction of causation using cross-sectional family data. Behav Genet. 1993;23:29–50.

16. Gillespie NA, Gehrman P, Byrne EM, Kendler KS, Heath AC, Martin NG. Modeling the direction of causation between cross-sectional measures of disrupted sleep, anxiety and depression in a sample of male and female Australian twins. J Sleep Res. 2012 Dec;21(6):675–83.

17. Webster GD, Dewall CN, Pond RS, Deckman T, Jonason PK, Le BM, et al. The brief aggression questionnaire: Psychometric and behavioral evidence for an efficient measure of trait aggression. Aggress Behav. 2014 Mar;40(2):120–39.

18. McDonald RP. Test theory: A unified treatment. psychology press; 2013.

19. Shrout PE, Fleiss JL. Intraclass correlations: uses in assessing rater reliability. Psychol Bull. 1979;86(2):420.

20. Rosseel Y. lavaan: An R package for structural equation modeling. J Stat Softw. 2012;48:1–36.
